# Drivers of engagement in virtual communities of practice: a qualitative study of Australian pharmacists’ perceptions and experiences

**DOI:** 10.1007/s11096-025-01913-3

**Published:** 2025-04-28

**Authors:** Abdella Birhan Yabeyu, Jo Cavanagh, Rachel Lawson, Kathy Le, Lili Schmah, Kenneth Lee, Deborah Hawthorne, Amy T. Page

**Affiliations:** 1 Department of Pharmacy, College of Medicine and Health Science, Ambo University, Ambo, Ethiopia; 2 Department of Pharmacy, School of Allied Health, University of Western Australia, Perth, Australia

**Keywords:** Community of practice, Focus groups, Pharmacist, Qualitative

## Abstract

**Background:**

In today’s digital age, virtual Communities of Practice allow pharmacists to connect and collaborate across geographical and professional boundaries. These platforms create opportunities for shared learning and knowledge exchange, fostering innovation and helping pharmacists stay informed about evolving practices.

**Aim:**

This study aimed to explore the factors that drive engagement in virtual Communities of Practice among Australian pharmacists.

**Method:**

A qualitative study was conducted with 24 Australian pharmacists recruited via social media, professional networks, and conferences. Five online focus groups (each with 3–4 participants) were conducted, lasting 45–60 min. Discussions were transcribed verbatim, and data were analysed thematically using the Framework Method. Rigor was ensured through convenience sampling, maintenance of an audit trail, and the use of independent coding and thematic analysis to enhance credibility and confirmability.

**Results:**

Four major themes emerged from the analysis: ‘access to information’, ‘sense of community’, ‘active facilitation’, and ‘platform usability’. Participants appreciated peer-shared knowledge and staying informed about current practices. A strong sense of community was cultivated as participants supported by others within the virtual community of practice. Active facilitation, such as moderators filtering content and engaging participants, was essential for maintaining a constructive environment. Additionally, platform usability, characterised by user-friendly features, the ability to keep personal and professional boundaries, and flexibility in accessing activities, significantly enhanced participant engagement.

**Conclusion:**

The study identified four key drivers of engagement in virtual Communities of Practice including accessible information, community support, active facilitation, and platform usability. These findings inform the design of virtual Communities of Practice to enhance professional collaboration and practice. Addressing these factors can enhance virtual Communities of Practice effectiveness in supporting professional collaboration, reducing isolation, and fostering continuous learning, particularly in evolving pharmacy roles.

**Supplementary Information:**

The online version contains supplementary material available at 10.1007/s11096-025-01913-3.

## Impact statements

- Participants in this study highlighted that Virtual Communities of Practices (vCoPs) are a key resource for accessing peer-reviewed, evidence-based information, supporting their ability to stay informed about legislative changes and best practices.
- The study findings indicate that pharmacists, particularly those in rural areas, rely on vCoPs to reduce professional isolation, build a sense of community, and collaborate with peers to enhance patient care.
- Participants emphasised that active facilitation within vCoPs was crucial for maintaining focused discussions and fostering constructive knowledge sharing, particularly in navigating new clinical responsibilities.
- The study found that pharmacists prefer vCoPs that integrate with familiar platforms, allowing flexible access and supporting sustained professional development.

## Introduction

Many pharmacists work in professionally isolated settings, often alone or in small teams [1]. Learning with and from peers helps professionals expand their knowledge and improve the quality of practice [2]. One strategy to address this isolation and foster a sense of community is the creation of virtual Communities of Practice (vCoPs) [3, 4]. vCoPs are groups of professionals who engage in collaborative learning and knowledge exchange through digital platforms, transcending geographical limitations. These can range from formal forums organised by professional bodies to informal professionals-run groups on social media platforms [5, 6].

Communities of Practice (CoPs) are groups of individuals with shared professional interests who engage regularly to improve their knowledge and skills [6]. The rise of digital technology has transformed these into vCoPs, allowing members to connect and collaborate beyond geographical limitations [3]. Key elements such as connectivity, leadership, and active participation are crucial for fostering a sense of community and promoting effective knowledge sharing within these groups [5]. Despite their benefits, CoPs often face challenges, such as low participation rates and engagement issues that can limit their effectiveness [7]. Addressing these issues requires identifying the key drivers of successful engagement. This study focuses on these drivers by exploring the unique experiences of Australian pharmacists in vCoPs. While effective CoPs employ strategies like regular meetings, collaborative knowledge sharing, and tailored resources to enhance engagement, these approaches remain underexplored in the context of pharmacy practice [8–10]. By addressing this gap, the study offers actionable insights for improving professional collaboration in vCoPs.

The role of pharmacists in Australia is expanding, with an increasing scope of practice, which is likely to enhance patient access to health services and improve health outcomes [11, 12]. Recent legislative changes have allowed pharmacists to prescribe medications and administer vaccinations, necessitating their adaptation to these new responsibilities [13]. In this context, mentorship and peer interaction through vCoPs are essential for overcoming professional isolation. Studies indicate that mentoring can effectively support pharmacists transitioning to these roles by bolstering their confidence and competence [14, 15]. Thus, the challenges faced in novel roles within often isolated work settings can be addressed by adopting vCoPs, which provide a network for staying updated with knowledge and facilitating role adjustment [2, 16, 17]. Previous studies on vCoPs in international healthcare and pharmacy contexts have mainly focused on professional collaboration, knowledge sharing, mentorship, and strategies for enhancing practice standards. While some of these studies explored participant engagement, their findings were often generalised and did not address the unique challenges faced by pharmacy practice in specific settings. Additionally, there is a lack of research specifically focusing on the engagement of pharmacists in vCoPs within the Australian context [18, 19]. Existing studies have either examined pharmacy-related vCoPs [1, 20, 21] or vCoPs within the broader Australian healthcare system but outside the pharmacy profession [7–10, 22–27].

Australian pharmacists face distinct challenges due to the rapid expansion of their professional responsibilities, driven by recent legislative reforms. This accelerated change, combined with the professional isolation common in rural and remote areas, highlights the need for support mechanisms [11, 13]. Current literature lacks insight into how these challenges uniquely affect pharmacists in Australia, particularly in fostering engagement and collaboration through vCoPs.

### Aim

This study aimed to explore the factors that drive engagement in vCoPs among Australian pharmacists, offering insights to optimise these platforms for enhanced professional collaboration and practice.

### Ethics approval

Ethical approval for the study was provided by the Human Ethics Office of the University of Western Australia by the requirements of the National Statement on Ethical Conduct in Human Research (National Statement) (ID: 2023/ET000283).

## Method

### Study design

A qualitative, descriptive methodology was employed through online focus groups to gain a comprehensive understanding of pharmacists'perceptions and experiences, specifically exploring the key factors driving their engagement in informal vCoPs. This study focused on pharmacist participation in social media-based communities, such as Facebook and LinkedIn groups. The Consolidated Criteria for Reporting Qualitative Research (COREQ) checklist was followed for reporting this study [28].

### Participants, recruitment, and sample

The inclusion criteria for the current study were Australian pharmacists and intern pharmacists licensed to practice in Australia with the Australian Health Practitioner Regulation Agency (AHPRA). Only participants who self-reported prior experience engaging in a vCoP were included. During recruitment, participants were asked to describe their engagement in a vCoP to confirm their eligibility. No minimum length of experience was required, but all participants had prior involvement in a vCoP. Those who did not meet this criterion were not included in the focus groups. We used convenience sampling, recruiting participants through virtual advertisements on LinkedIn and existing pharmacy-related vCoPs on Facebook. Participants were also recruited in person at the Pharmaceutical Society of Australia’s 2023 national, annual conference and through the researchers’ professional networks.

### Data collection procedure

Data were collected through virtual focus group discussions conducted over three in August 2023. Each group consisted of three to four participants, and the sessions were facilitated via Microsoft Teams until data saturation was achieved, with no new codes emerging after two consecutive group discussions, as determined by the research team. The focus group format was selected for its interactive and collaborative nature, which enabled the exchange of diverse perspectives and experiences [29, 30].

A semi-structured focus group discussion guide was developed and pilot-tested through role-play among the researchers, with subsequent adjustments applied to the guide. The guide explored key topics such as motivations for engaging in vCoPs, perceived benefits and challenges, preferred engagement strategies, and platform usability (Supplementary File 1). At least three researchers attended each focus group to record audio and video and take field notes.

### Data analysis

The collected data were transcribed verbatim using online transcription software (Otter.ai). The accuracy of the transcription was then manually verified, and any errors were corrected before analysis. We used the Framework Method [31] a systematic approach to qualitative data analysis, and NVivo software was used to conduct the thematic analysis. Data analysis followed an inductive thematic approach, allowing themes to emerge directly from the data. Transcripts were coded inductively, and the responses were categorised according to the established framework to derive insights and relationships pertinent to the research questions.

### Quality assurance

The interview guide was piloted two weeks before data collection through a role-play session conducted among the research team. This process involved mock interviews where researchers acted as both facilitators and participants to assess the clarity, flow, and relevance of the questions. Feedback from these sessions was discussed among the research team, leading to minor modifications to enhance question clarity and ensure alignment with the study objectives. To ensure the study’s trustworthiness, several strategies were employed. Confirmability was achieved through analyst triangulation, where independent coding of the data was carried out by multiple researchers. Discrepancies in coding were discussed and resolved collaboratively, enabling an unbiased interpretation of the findings. Credibility was further enhanced through peer debriefing, with researchers engaging in ongoing discussions to confirm the accuracy of data interpretation. In addition, an audit trail was maintained to document key methodological decisions, which supports the dependability of the study. Finally, the detailed description of participant characteristics and the study context provides insight into the transferability of the findings to other settings [32].

## Results

### Sociodemographic characteristics of the study participants and focus group summary

A total of 45 pharmacists initially expressed interest in the study, and 24 participated in five focus groups. The remaining 21 pharmacists did not participate primarily due to not meeting the inclusion criterion of prior experience in a vCoP. Additionally, some withdrew due to scheduling conflicts. Data from these 24 participants were included in the final analysis. Focus groups lasted between 45 and 60 min. The participants represented a diverse range of experiences and attributes, as indicated by the demographic information collected during registration (Table 1). Data analysis revealed four major themes: ‘access to information,’ ‘sense of community,’ ‘active facilitation,’ and ‘platform usability.’ Within these major themes, eight subthemes were identified (Table 2).

**Table 1:** Demographics Information of the study participants

	All Participants, n (%)
Total number of participants	24 (86%)
*Gender*	
Men	4 (16.7%)
Women	20 (83.3%)
*Age (years)*	
20–29	11 (45.8%)
30–39	9 (37.5%)
40–49	3 (12.5%)
50+	1 (4.2%)
*Location*	
ACT	2 (8.4%)
NSW	2 (8.4%)
QLD	6 (25.0%)
VIC	1 (4.2%)
WA	13 (54.2%)
*Geographical setting*	
Metropolitan	16 (66.7%)
Regional	3 (12.5%)
Rural	4 (16.7%)
Remote	1 (4.2%)
*Years of practice*	
0 (intern)	2 (8.3%)
1–4	10 (41.7%)
5–9	4 (16.7%)
10–14	3 (12.5%)
15 +	5 (20.8%)
*Current hours worked per week*	
0	2 (8.3%)
1–19	3 (12.5%)
20–39	8 (33.3%)
40–59	10 (41.7%)
60 +	1 (4.2%)
*Pharmacy setting experience*	
*Community*	21 (75%)
Hospital	8 (29%)
Home medicines reviews (HMR)	6 (21%)
*Residential medicine* Management reviews	2 (7%)
*General practice*	2 (7%)
Research	3 (11%)
Academia	2 (7%)
Industry	2 (7%)
Government	1 (4%)
Compounding	0

*ACT* australian capital territory, *NSW* New South Wales, *QLD* Queensland, *VIC* Victoria, *WA* Western Australia

**Table 2:** Summary of major themes, subthemes, and additional participant quotes identified through data analysis to complement textual findings

Major themes	Subthemes	Examples of participant quotes
Access to information	Learning from other pharmacists	*I think when I first joined [a pre-existing vCoP], I didn't know I wanted to pursue the accreditation process. And when I joined that page, and I saw how much people were interacting, and just how interesting the case studies that they were getting… and it just highlighted the process as well, which was quite vague at the time. So, I think it just illustrated how much more I could possibly do to further my career.” (Participant 11, Community and Home Medicines Review pharmacist, 10 years of practice)*
	Keeping up to date with information	*I think we're always going to need someone to kind of help synthesise and condense some of that information. That's so beneficial than trying to muddle through it yourself, individually. It's really helpful to have those dedicated conversations about changing legislation and, and that sort of stuff. And to know where you can get some information about that that's going to be “okay, what does this actually mean for pharmacy?” Not, I guess ‘politicians speak’ in terms of, you know, news items, and that sort of stuff, determining what it actually means for pharmacy. So, I think we are going to need that because it’s changing every single day.” (Participant 13, community pharmacist, one year of practice)*
Sense of Community	Experiencing support	*Sometimes it's just sharing the experience and knowing that you're not the only one going through that situation.” (Participant 1, community pharmacist, 22 years of practice)*
Active Facilitation	Facilitator filtering content	*I think [the role of the facilitator] is just ensuring that discussion and comments are always respectful and appropriate, which is so important especially when there's so much going on in the world of pharmacy, and perhaps a lot of frustration from, from people. That yeah, it's important that the environment feels supportive and safe to contribute discussion.” (Participant 23, GP pharmacist, 12 years of practice)*
	Facilitator engaging group members	*I think it's very important to have a good moderator who is a good communicator and can be engaging with people, can sort of encourage good discussions, bring up topics that will sort of get people thinking and, and get people commenting. Nothing too outlandish, but just someone who will really truly engage, you know, what's happening and around and be also sort of very knowledgeable.” (Participant 22, community and HMR pharmacist, 40 years of practice)*
Platform Usability	Maintaining personal and professional boundaries	*Definitely virtual and online meetups. I think since the last three years, people are kind of adjusted to that. And it's not as bad as it used to be. And also, more convenient. I mean, for me to jump on this at eight in the evening. It's [Australian Eastern Standard] time. Easy. If you had to tell me to go into the city, and meet people, that's half an hour travel going, half an hour coming back, and that's two hours of my evening gone.” (Participant 24, hospital and HMR pharmacist, 25 years of practice)*
	Access to online activities	*Being able to support each other on a regular, or meet each other on a weekly basis, I would say. I think just to have that frequency to be able to stay in touch and connected to this, like virtual, online community.” (Participant 6, RMMR and compounding pharmacist, six years of practice)*
	Easily accessible content	*They should make different subgroups within the group. So, you can sort by the type of post there is because sometimes I don't really need to hear about just random funny things I don't really care about because I'm mostly there for information. So, if they sort that out by subgroups, it'd be amazing. So, you can skip the ones you don't need. In a more efficient way. That'd be good.” (Participant 2, community pharmacist, one year of practice)*

### Access to information

Findings indicate that access to shared information within the vCoP is a crucial factor and a key driver for engagement. Participants emphasised that the ability to view peer-shared knowledge and incorporate updated information into their professional practices made the vCoP indispensable. The ease of access to useful, relevant, and current information emerged as a significant motivator for engagement. Within this theme, subthemes emerged, focusing on learning from other pharmacists and staying up-to-date with information.

### Learning from other pharmacists

Participants stated that the ability to access knowledge and experiences shared by fellow pharmacists was not only beneficial but also a pivotal factor in fostering engagement within the vCoP. Learning from peers enabled participants to gain diverse perspectives and apply this knowledge to their professional practice, motivating continued participation in the community. The prospect of continuous professional development emerged as a significant driver for sustained engagement. Participants valued considering different viewpoints shared by other pharmacists, including accessing expertise from those with varied backgrounds. This coupled with their ability to share and hear others’ experiences, facilitated valuable information exchange and further learning within the group.

*“Seeing things from different perspectives has been great for me, like when people have posted,...how would you respond to this? Or we had this scenario, what would you do? Obviously, I know my way of kind of thinking, and then when I was an intern, I had my preceptors'way of thinking and things like that, but then, like reading 50 different opinions has been great for me, like developmentally, because then I can go, hey, I didn’t actually think of, you know, responding like that, or things like that.” (Participant 8, community & hospital pharmacist, 3 years of practice)*

*“I quite like sometimes in groups I'm in, we kind of share just an interesting case from our day, or our week or since last time since we caught up. So, you might have had an interesting scenario or a really challenging scenario, and sharing that, getting perhaps advice from the group or just sharing it for their learning too, of the process that was involved.” (Participant 23, General Practice pharmacist, 12 years of practice).*

Participants also appreciated the opportunity to access information and networks that had the potential to be used for career progression. This was achieved through learning of how others had progressed through their careers and by connecting with others within the groups who could assist with achieving personal career goals.

### Keeping up to date with information

Participants appreciated the ability to access current information, which was highlighted as essential for informing best practices among pharmacists across various sectors. The opportunity to consolidate information allowed them to gain a clearer understanding of legislative changes, which emerged as a key advantage. Accessing reliable, evidence-based information and having easy access to this knowledge were particularly important, as they enabled participants to apply what they learned in their workplaces. This continuous access to relevant information not only enhanced their professional practice but also served as a significant driver for sustained engagement in the vCoP, motivating them to remain active members of the community.

*“I think definitely keeping a hand in clinically, like reading the case studies is really interesting for me, and it sort of keeps me up to date with the latest evidence. But then also might be on any topic from like medicine shortages or evidence for a particular drug to treat a particular condition, you can sort of take that back into the workplace. And maybe it's been a couple of times, I've actually sort of been out to apply what someone might have done in a post or refer to like, the latest guidelines. And then I think, oh, yes, I remember that post. I'll go back to it, and then have a look at those guidelines and be able to apply it in my workplace.” (Participant 19, Government pharmacist, four years of practice).*

### Sense of community

This theme highlights participants'perceived value in cultivating a sense of community and acceptance within the vCoP. Participants reported that a strong communal identity significantly enhanced their motivation to engage, as it fostered a supportive environment that facilitated a sense of connection with other pharmacists. Within this theme, a subtheme emerged regarding participants experiencing support. This atmosphere of belonging and mutual support enriched their professional relationships and encouraged ongoing participation in vCoPs, rendering them more inclined to contribute actiely and share experiences.

#### Experiencing support

Participants appreciated the support and connection they received from others in the group. This support was vital in mitigating the professional isolation they faced, enabling them to engage with colleagues with whom they had previously limited interactions. The diminishing feelings of isolation, whether geographical or stemming from expertise or structural barriers, made participants feel more empowered to actively participate in vCoPs.

*“For me, it was a connection as well and networking in the context of, I guess, in my role and where I am, there's no other pharmacist in [name of a rural town in Australia], which is where I am, that is in a general practice role or were at the time. So, to be able to connect with others doing the same role it helped, I guess, reduce the feeling of isolation in my work.” (Participant 23, GP pharmacist, 12 years of practice).*

Participants also reported experiencing support through connections with others who had shared similar experiences. This led to feelings of validation and comradery amongst members.

*“I do get that satisfaction of knowing that, especially in [an already existing vCoP], a lot of people just post how they feel. And sometimes that can bring you down but sometimes, it's also comforting to know you're not the only one feeling this way. And you feel like you're a part of a larger community.” (Participant 19, community pharmacist, one year of practice).*

### Active facilitation

Participants perceived the facilitator's role as highly valuable in their vCoP experience, particularly when facilitators were actively engaged and proactive. Such an active facilitation approach ensured that discussions remained relevant and focused, fostering a collaborative environment that encouraged member participation in vCoP. Subthemes within this theme included the facilitator's role in filtering content and engaging group members in discussions. By promoting interaction and sustaining a constructive atmosphere, facilitators played a pivotal role in driving engagement within the community.

#### Facilitator filtering content

Participants emphasised the importance of proactive facilitation in filtering content within the vCoP to align with group goals. This included the facilitator editing content to ensure relevance in discussions to align with the group purpose and editing content to ensure respect between participants was consistently maintained.

*“[the facilitator] needs to be proactive and be the mediator and make sure that all the discussions are appropriate and related to the pharmacy industry.” (Participant 9, community and hospital pharmacist, 11 years of practice).*

#### Facilitator engaging group members

Participants expressed it to be valuable for facilitators to also engage group members in the content shared within vCoPs. Participants preferred when the facilitator would engage members by encouraging them to actively participate in the content shared to ensure multiple members expressed their thoughts and contributed to the discussions. Additionally, participants appreciated facilitators prompting discussions within the group, through initiating conversations for members to subsequently engage in.

### Platform usability

This theme reflects the effective deployment of platforms facilitating vCoPs, ensuring they fulfill their intended purpose and structure. Subthemes within platform usability encompass the ability to maintain personal and professional boundaries, being able to access online activities, and ensuring easily accessible content. By providing a user-friendly environment that respects the privacy and promotes interaction, these platforms enhance member engagement and foster active participation in the vCoP.

#### Maintaining personal and professional boundaries

Participants indicated the importance of vCoPs being accessible in ways that respected their personal and professional preferences. This included maintaining appropriate boundaries through the vCoP platform, with features that enabled participants to experience their desired level of privacy and flexibility while participating in the group. Upholding personal and professional boundaries facilitated greater comfort among participants when engaging in discussions and sharing their experiences, ultimately enhancing their overall engagement within the vCoP.

*“I'd say, as healthcare professionals, privacy is very important. It's not just the privacy of the patients or communities but also the privacy of the businesses, the pharmacies and the practitioners ourselves. So, I think most people are very aware of it.” (Participant 7, community pharmacist, nine years of practice).*

Regarding flexibility, it was noted as important and useful for pharmacists to be able to access a vCoP platform in ways that were most convenient to them. This included flexibility around when they chose to access the group and flexibility in how they could access the group, such as having the CoP online, instead of face-to-face.

#### Access to online activities

Participants valued having access to platforms that facilitated online activities. This included the ability of the platform to host regular meetings and allow users to easily view and participate in discussions on relevant topics. Access to these online activities provided opportunities for learning and collaboration and fostered a sense of community, encouraging participants to engage more actively in the vCoP.

#### Easily accessible content

Participants emphasised the importance of having content shared within vCoPs in ways that were easy to use and aligned with group goals. A key consideration for participants was the simplicity of access, particularly the ability to engage with vCoPs through familiar platforms they were already using. This preference meant they did not have to create new logins or learn to navigate unfamiliar platforms, which could deter participation. Participants ensure that content is easily accessible, vCoPs facilitate greater member engagement, allowing participants to focus on collaboration and learning rather than technical barriers.

*“It needs to be kind of integrated into what you're already using, in my opinion, rather than having to have another login, another thing to check… it gets a little bit overwhelming, and you kind of only channel like one or two things at a time and focus on it. So, you don't want it to get forgotten about. So whatever platform is used, it kind of needs to be integrated into what we already have somehow.” (Participant 21, community pharmacist, three years of practice).*

Communicating in clear and professional ways when accessing content was also noted as important by participants. Pharmacists expressed a preference for communication that was clear, easily understood, and reflective of their professional status.

*“One thing for me, is you need to actually form proper sentences…you need to actually type properly to communicate effectively. And I think like in a pharmacist group, that’s especially important because we’re all health professionals, we’re all well-educated. So, we need to be able to communicate effectively, I guess, in that kind of way. Because in other groups that I’m in, that are not pharmacists related, sometimes you’re reading the posts, and it’s like, I actually have no idea what you’re talking about, can be kind of frustrating.” (Participant 8, community and hospital pharmacist, three years of practice).*

The formation of ‘subgroups’ within the platform used was noted to be of value to participants also. This was identified as an element through which content was most accessible, as it allowed users to be able to view and participate in smaller groups, which were focused on topics most relevant to each participant. To provide additional context and participant opinion while adhering to word count constraints, Table 2 includes supplementary illustrative quotes that complement the themes and subthemes discussed in the text.

## Discussion

This study explored key factors driving engagement among Australian pharmacists in informal vCoPs hosted on social media platforms (e.g., Facebook, LinkedIn), identifying four primary drivers: access to information, sense of community, active facilitation, and platform usability. These elements support collaborative learning, participation, and the accessibility of relevant content, aligning with the study’s objective of understanding what motivates Australian pharmacists to engage in vCoPs. Participants engaged in multiple pharmacist-related vCoPs, which varied in size, purpose, and level of interaction. Some groups fostered active engagement with daily discussions, while others were primarily passive information-sharing platforms, where members consumed content without contributing regularly.

Previous Australian studies have underscored the benefits of pharmacist-led vCoPs in fostering professional collaboration, mentoring, and ongoing development [12]. For instance, a prior study has examined how pharmacist vCoPs facilitate the exchange of knowledge on prescribing practices and their role in peer mentoring and support for early-career pharmacists [21]. The study findings build upon this existing literature by highlighting the crucial function of social media-based vCoPs, which allow pharmacists to surmount geographic and organisational barriers, access up-to-date information, and cultivate professional networks in a flexible, easily accessible environment.

The findings from this study extend beyond the Australian context, providing a broader understanding of how vCoPs functions globally across healthcare professions. These insights can inform how different healthcare systems leverage vCoPs to support professional learning, knowledge-sharing, and interprofessional collaboration. The following sections discuss how these findings relate to existing literature and their implications for advancing the pharmacy profession.

Access to date and peer-reviewed information has emerged as a crucial factor driving pharmacists’ engagement in vCoPs. Participants emphasised the importance of quickly accessing relevant, evidence-based content to support their professional practices. This aligns with previous studies highlighting the significance of information sharing and accessibility in healthcare-focused virtual communities [25, 26]. Furthermore, pharmacists value vCoPs for facilitating continuous professional development by providing current, relevant, and easily understandable content, which is vital in the evolving pharmacy profession, particularly regarding legislative changes and clinical best practices [33]. To enhance engagement, vCoPs must ensure their content is reliable, tailored to the practical needs of pharmacists, and promotes sustained participation and professional growth [22, 26].

A strong sense of community plays a critical role in driving engagement in vCoPs among Australian pharmacists. Participants noted that receiving professional support from peers was essential for overcoming the inherent isolation often experienced in pharmacy practice, especially in rural or remote areas [34, 35]. This sense of belonging facilitated more frequent knowledge exchange and collaboration, which participants valued for advancing their careers and staying current with clinical practices [25, 26]. The study findings highlight its distinctive significance in the Australian pharmacy context, where geographically isolated professionals heavily depend on virtual platforms for support. This highlights the necessity of designing vCoPs with features that promote a sense of community, thereby enhancing engagement and professional development [3].

Active facilitation is crucial for driving engagement within vCoPs among Australian pharmacists. Facilitators who proactively moderate discussions, filter content, and encourage member participation significantly enhance the relevance and focus of the discussions [8]. This approach fosters a collaborative environment that sustains constructive dialogue and increases participation. Moreover, facilitators who actively engage members ensure a wider range of voices contribute to the discussions. While previous studies have highlighted the role of facilitation in vCoPs [8, 24, 34], these study also emphasises its particular importance for Australian pharmacists, who face unique challenges in accessing timely information and collaborative learning opportunities essential for adapting to their expanding roles in a rapidly evolving profession [6, 33].

The study found that platform usability is crucial for pharmacists'engagement in vCoPs. Pharmacists expressed a preference for platforms that offer flexible access, allowing participation at convenient times and through familiar interfaces. While a previous study has highlighted privacy concerns and emphasised the importance of usability in healthcare-based virtual communities [33], this study adds that pharmacists favor platforms that seamlessly integrate into their professional workflows and are easy to navigate. To enhance engagement, vCoPs should prioritise user-friendly designs that respect professional boundaries while ensuring content is accessible and relevant. These findings have significant implications for the future development of vCoPs, suggesting that improved usability could foster sustained participation and support professional growth [30]. Although the study was undertaken in 2023, the participants did not directly attribute their engagement in vCoPs to the COVID-19 pandemic. Nevertheless, the indirect influence of the pandemic on legislative modifications and digital adaptation may have contributed to pharmacists'heightened dependence on virtual platforms. Prospective studies could examine the long-term implications of the pandemic on vCoP participation more explicitly.

### Limitations and strengths

This study had several limitations. The participant pool primarily consisted of urban and metropolitan pharmacists, with limited rural and remote representation, despite these groups potentially benefiting the most from vCoPs. The sample spanned diverse experience levels, but the impact of career stage on vCoP engagement was not systematically explored. Furthermore, the study did not collect data on the size and scope of participants'vCoPs, which may have constrained the ability to fully contextualise engagement patterns. These factors may have introduced selection bias and limited the generalisability, particularly for underrepresented groups like rural pharmacists.

Despite these limitations, this study offers valuable insights into the factors driving Australian pharmacists'engagement in vCoPs. A rigorous qualitative methodology was conducted to ensure trustworthiness. Confirmability was established through an audit trail that documented key methodological decisions. Credibility was enhanced through analyst triangulation, involving independent coding and consensus discussions among researchers to resolve discrepancies. Transferability was supported by including pharmacists from diverse regions and sectors across Australia, enriching the contextual relevance of the findings. Finally, dependability was ensured through the systematic application of the Framework Method for data analysis, providing a clear and replicable approach. These strengths collectively reinforce the trustworthiness of this study, offering a deeper understanding of the factors driving pharmacist engagement in vCoPs and providing valuable insights for optimising these platforms to support professional practice.

### Further research

Further research is needed to explore the effective utilisation of vCoPs among Australian pharmacists. Future studies should focus on strategies for mentoring members, facilitating engagement, and achieving a balance between formality and informality. Examining the motivating factors that drive pharmacists to participate in vCoPs could also enhance adoption rates and user satisfaction. Additionally, longitudinal studies would offer valuable insights into the long-term viability of vCoPs and their potential to improve pharmacy practice. Future research should explore vCoP characteristics, including membership, accessibility, and participation duration, to understand engagement and sustainability. Investigating inactive vCoPs could provide insights into challenges hindering their longevity, informing strategies to ensure the effectiveness of these collaborative spaces.

## Conclusion

The analysis identified several key drivers of engagement within vCoPs for Australian pharmacists: access to reliable information, a strong sense of community, active facilitation, and platform usability. While these findings provide valuable insights, additional factors may emerge in studies with larger or more diverse participant groups. These factors play a crucial role in motivating pharmacists to participate actively in vCoPs, promoting shared learning and professional growth. Insights from this study guide optimising both new and existing pharmacy related vCoPs to enhance engagement and collaborative knowledge exchange. By addressing these engagement drivers, Australian pharmacy practice can benefit from more cohesive, informed, and connected professional networks, ultimately improving patient care and pharmacy practice outcomes.

## Supplementary Information

Below is the link to the electronic supplementary material.

Supplementary file1 (DOCX 17 KB)

Supplementary file2 (PDF 97 KB)

Supplementary file3 (PDF 83 KB)

Supplementary file4 (PDF 79 KB)

Supplementary file5 (PDF 99 KB)

## References

[1] Guénette L, Maheu A, Vanier M-C, et al. Pharmacists practising in family medicine groups: an evaluation 2 years after experiencing a virtual community of practice. Can Pharm J. 2022;155:39–49. 10.1177/17151635211049235. 10.1177/17151635211049235 PMC8756373 35035641

[2] McLoughlin C, Patel KD, O’Callaghan T, et al. The use of virtual communities of practice to improve interprofessional collaboration and education: findings from an integrated review. J Interprof Care. 2018;32:136–42. 10.1080/13561820.2017.1377692. 29161155 10.1080/13561820.2017.1377692

[3] Haas A, Abonneau D, Borzillo S, et al. Afraid of engagement? Towards an understanding of engagement in virtual communities of practice. Knowl Manag Res Pract. 2021;19:169–80. 10.1080/14778238.2020.1745704.

[4] Ranmuthugala G, Plumb JJ, Cunningham FC, et al. How and why are communities of practice established in the healthcare sector? A systematic review of the literature. BMC Health Serv Res. 2011;11:273. 10.1186/1472-6963-11-273. 21999305 10.1186/1472-6963-11-273 PMC3219728

[5] Wenger E. Communities of practice. 1st ed. Cambridge: Cambridge University Press; 1998. ISBN: 978-0521663632. 10.1017/CBO9780511803932.

[6] Wenger E, McDermott R, Snyder WM. Seven principles for cultivating communities of practice. In: Wenger E, McDermott R, Snyder WM, editors. Cultivating communities of practice: a guide to managing knowledge. Boston: Harvard Business Review Press; 2002. pp. 1–9. ISBN: 978–1578513307. 10.1177/875697280203300312.

[7] García-Monge A, González-Calvo G, Bores-García D. ‘I like the idea but…’: the gap in participation in a virtual community of practice for analysing physical education. J Open Distance e-Learn. 2019;34:257–72. 10.1080/02680513.2018.1505486.

[8] Denholm JT, Johnson D, Denholm GC, et al. Relational Gestalt approach to building a COVID-19 community of practice in a hospital setting. BMJ Leader. 2022;6:243–5. 10.1136/leader-2021-000587. 36170480 10.1136/leader-2021-000587

[9] Mullan J, Metusela C, Guppy M, et al. Development of a COVID-19 virtual community of practice in New South Wales: a qualitative study. Aust J Gen Pract. 2022;51:263–9. 10.31128/AJGP-03-21-5881. 35362011 10.31128/AJGP-03-21-5881

[10] Wilson A, Cornett M, Delbridge R, et al. A realist evaluation of a community of practice for dietitians and nutritionists working in Aboriginal and Torres Strait Islander health. J Hum Nutr Diet. 2023;36:277–87. 10.1111/jhn.13043. 35614859 10.1111/jhn.13043 PMC10084382

[11] Taylor S, Cairns A, Glass B. Health professional perspectives of expanded practice in rural community pharmacy in Australia. Int J Pharm Pract. 2020;28:458–65. 10.1111/ijpp.12648. 32602603 10.1111/ijpp.12648

[12] Sudeshika T, Deeks LS, Naunton M, et al. Evaluating the potential outcomes of pharmacist-led activities in the Australian general practice setting: a prospective observational study. Int J Clin Pharm. 2023;45:980–8. 10.1007/s11096-023-01604-x. 37269443 10.1007/s11096-023-01604-x PMC10239215

[13] Patel C, Vette K, Dalton L, et al. Assessment of the first 5 years of pharmacist-administered vaccinations in Australia: learnings to inform expansion of services. Public Health Res Pract. 2024;34:1–12. 10.17061/phrp3432420. 10.17061/phrp3432420 39443092

[14] Fowler JL, McConachie B, Hattingh L, et al. Mentoring pharmacy staff to implement a medication support service: an evaluation of process and outcomes. Curr Pharm Teach Learn. 2018;10:886–94. 10.1016/j.cptl.2018.04.004. 30236425 10.1016/j.cptl.2018.04.004

[15] Farrell B, Dolovich L, Austin Z, et al. Implementing a mentorship program for pharmacists integrating into family practice: practical experience from the IMPACT project team. Can Pharm J. 2010;143:28–36. 10.3821/1913-701X-143.1.28.

[16] Department of Health. Australian Government Response to the Final Report of the Royal Commission into Aged Care Quality and Safety. Canberra: Department of Health, 1 March 2021. ISBN: 978-1-76007-435-7. Accessed: 17 November 2024.

[17] Butler M. Minister for Health and Aged Care – speech – Pharmaceutical Society of Australia Conference – 28 July 2023 | Health Portfolio Ministers | Australian Government Department of Health and Aged Care. Available from: https://www.health.gov.au/ministers/the-hon-mark-butler-mp/media/minister-for-health-and-aged-care-speechpharmaceutical-society-of-australia-conference-28-july-2023. 2023; 1–13. Accessed: 21 November 2024.

[18] Taylor S, Cairns A, Glass B. Expanded practice in rural community pharmacy in Australia: pharmacists’ perspectives. J Pharm Pract Res. 2021;51:43–53. 10.1002/jppr.1688.

[19] Tan ECK, Stewart K, Elliott RA, et al. Integration of pharmacists into general practice clinics in Australia: the views of general practitioners and pharmacists. Int J Pharm Pract. 2014;22:28–37. 10.1111/ijpp.12047. 23750666 10.1111/ijpp.12047

[20] Lee K, Etherton-Beer C, Johnson J, et al. Utilising a ‘community of practice’ to support pharmacists to work in residential aged care: protocol for a longitudinal evaluation. BMJ Open. 2024;14:e076856. 10.1136/bmjopen-2023-076856. 38740504 10.1136/bmjopen-2023-076856 PMC11097797

[21] Bucknell L, Chambers B, Nott S, et al. Community pharmacists’ perceptions of a hospital based virtual clinical pharmacy service: findings from qualitative research. Explor Res Clin Soc Pharm. 2024;14:100437. 10.1016/j.rcsop.2024.100437. 38660625 10.1016/j.rcsop.2024.100437 PMC11040165

[22] Barnett S, Jones SC, Bennett S, et al. Perceptions of family physician trainees and trainers regarding the usefulness of a virtual community of practice. J Med Internet Res. 2013;15:e92. 10.2196/jmir.2555. 23666237 10.2196/jmir.2555 PMC3650926

[23] Wilding C, Curtin M, Whiteford G. Enhancing occupational therapists’ confidence and professional development through a community of practice scholars. Aust Occup Ther J. 2012;59:312–8. 10.1111/j.1440-1630.2012.01031.x. 22934904 10.1111/j.1440-1630.2012.01031.x

[24] Metzger MJ, Dick J, Gardner A, et al. Knowledge sharing, problem solving and professional development in a Scottish ecosystem services community of practice. Reg Environ Change. 2019;19:2275–86. 10.1007/s10113-019-01537-0.

[25] Moore JL, Bjørkli C, Havdahl RT, et al. A qualitative study exploring contributors to the success of a community of practice in rehabilitation. MC Med Edu. 2021;21:282. 10.1186/s12909-021-02711-x. 10.1186/s12909-021-02711-x PMC8130156 34001073

[26] Sibbald SL, Burnet ML, Callery B, et al. Building a virtual community of practice: experience from the Canadian foundation for healthcare improvement’s policy circle. Health Res Policy Syst. 2022;20:95. 10.1186/s12961-022-00897-0. 36050686 10.1186/s12961-022-00897-0 PMC9434556

[27] Silverstein A, Benson A, Gates C, et al. Global community of practice: a means for capacity and community strengthening for health professionals in low- and middle-income countries. J Glob Health. 2022;12:1–9. 10.7189/jogh.12.04034. 10.7189/jogh.12.04034 PMC9107096 35567589

[28] Tong A, Sainsbury P, Craig J. Consolidated criteria for reporting qualitative research (COREQ): A 32-item checklist for interviews and focus groups. Int J Qual Health Care. 2007;19:349–57. 10.1093/intqhc/mzm042. 17872937 10.1093/intqhc/mzm042

[29] Guest G, Namey E, O’Regan A, et al. Comparing interview and focus group data collected in person and online. Patient-Centered Outcomes Res Inst. 2020;19:1–62. 10.25302/05.2020.ME.1403117064. 36701499

[30] Guest G, Namey E, Taylor J, et al. Comparing focus groups and individual interviews: findings from a randomized study. Int J Soc Res Methodol. 2017;20:693–708. 10.1080/13645579.2017.1281601.

[31] Gale NK, Heath G, Cameron E, et al. Using the framework method for the analysis of qualitative data in multi-disciplinary health research. BMC Med Res Methodol. 2013;13:117. 10.1186/1471-2288-13-117. 24047204 10.1186/1471-2288-13-117 PMC3848812

[32] Ali AMd, Yusof H. Quality in qualitative studies: the case of validity, reliability and generalizability. Issues Soc Environ Account. 2011;5:1–41. 10.22164/isea.v5i1.59.

[33] Rolls K, Hansen M, Jackson D, et al. How health care professionals use social media to create virtual communities: an integrative review. J Med Internet Res. 2016;18:1–19. 10.2196/jmir.5312. 10.2196/jmir.5312 PMC4933801 27328967

[34] Barnett S, Jones SC, Bennett S, et al. General practice training and virtual communities of practice: a review of the literature. BMC Fam Pract. 2012;13:1–12. 10.1186/1471-2296-13-87. 22905827 10.1186/1471-2296-13-87 PMC3515460

[35] Rolls KD, Hansen MM, Jackson D, et al. Why health care professionals belong to an intensive care virtual community: qualitative study. J Med Internet Res. 2019;21:1–16. 10.2196/14068. 10.2196/14068 PMC6864486 31687936

