# Supplementary material for: Drivers of engagement in virtual communities of practice: a qualitative study of Australian pharmacists’ perceptions and experiences
Source: Int J Clin Pharm. 2025 Apr 28;47(5):1286–95. doi: 10.1007/s11096-025-01913-3 (PMC12431881; doi:10.1007/s11096-025-01913-3)
Supplement: Supplementary file 1 — Supplementary file1 (DOCX 17 KB) [file 11096_2025_1913_MOESM1_ESM.docx]

**Appendix 1: COREQ Checklist**

| No. | Item | Guide questions/description | Detail reported/section of manuscript reported in |
| --- | --- | --- | --- |
| DOMAIN 1: RESEARCH TEAM AND REFLEXIVITY | | | |
| Personal characteristics | | | |
| 1 | Interviewer/facilitator | Which author/s conducted the interview or focus group? | DH and RL facilitated the focus groups. See Methods – Focus group facilitation |
| 2 | Credentials | What were the researcher’s credentials? *Eg. PhD, MD* | Student researchers have no prior credentials |
| 3 | Occupation | What was their occupation at the time of the study? | JC, RL, KL and LS – Master of Pharmacy students at UWA |
| 4 | Gender | Was the researcher male or female? | Primary researchers were all female |
| 5 | Experience and training | What experience did the researcher have? | Student researchers had no experience |
| RELATIONSHIP WITH PARTICIPANTS | | | |
| 6 | Relationship established | Was a relationship established prior to study commencement? | Some participants may have known researchers and facilitators because participants were recruited through professional networks |
| 7 | Participant knowledge of the interviewer | What did the participant know about the researcher? *Eg. Personal goals, reason for doing the research* | Reason for doing the research and occupation |
| 8 | Interviewer characteristics | What characteristics were reported about the interviewer/facilitator? | Occupation |
| DOMAIN 2: STUDY DESIGN | | | |
| THEORETICAL FRAMEWORK | | | |
| 9 | Methodological framework and theory | What methodological orientation was stated to underpin the study? *Eg.* *Grounded theory, discourse analysis, ethnography, phenomenology, content analysis* | Qualitative descriptive method using the framework method. See Methods – study design |
| PARTICIPANT SELECTION | | | |
| 10 | Sampling | How were participants selected? *eg. Purposive, convenience, consecutive, snowball* | Voluntary response sampling – see Methods - recruitment |
| 11 | Method of approach | How were participants approached? *Eg. Face-to-face, telephone, mail, email* | Via social media and face-to-face. See Methods – recruitment |
| 12 | Sample size | How many participants were in the study? | 28. See Results |
| 13 | Non-participation setting | How many people refused to participate or dropped out? Reasons? | None |
| SETTING | | | |
| 14 | Setting of data collection | Where was the data collected? *Eg. Home, clinic, workplace* | Online via Microsoft Teams video conference – See Methods – Study design |
| 15 | Presence of non-participants | Was anyone else present besides the participants and researchers? | No |
| 16 | Description of sample | What are the important characteristics of the sample? *eg. Demographic data, date* | See demographics table (Table 1) |
| DATA COLLECTION | | | |
| 17 | Interview guide | Were questions, prompts, guides provided by the authors? Was it pilot tested? | Yes – See methods – study design and Link 1 for complete interview guide |
| 18 | Repeat interviews | Were repeat interviews carried out? If yes, how many? | No |
| 19 | Audio/visual recording | Did the research use audio or visual recording to collect the data? | Both were recorded but only audio was used for analysis – See Methods – Focus group facilitation |
| 20 | Field notes | Were field notes made during and/or after the interview or focus group? | Yes. See Methods – focus group facilitation |
| 21 | Duration | What was the duration of the interviews or focus group? | 45 minutes to 1 hours. See Methods – study design |
| 22 | Data saturation | Was data saturation discussed? | Yes. See Methods – data analysis |
| 23 | Transcripts returned | Were transcripts returned to participants for comment and/or correction? | No |
| DOMAIN 3: ANALYSIS AND FINDINGS | | | |
| DATA ANALYSIS | | | |
| 24 | Number of data coders | How many data coders coded the data? | Two – LS and RL |
| 25 | Description of the coding tree | Dis authors provide a description of the coding tree | Yes – See supplementary links 2, 3 & 4 for coding tree, code book and audit trail |
| 26 | Derivation of themes | Were themes identified in advanced or derived from the data? | Derived from the data. See Methods – data analysis |
| 27 | Software | What software, if applicable was used to manage the data? | NVivo coding software. See Methods- data analysis |
| 28 | Participant checking | Did participants provide feedback on the findings? | No |
| REPORTING | | | |
| 29 | Quotations presented | Were participants quotations presented to illustrate the themes/findings? Was each quotation identified? Eg. Participant number | Yes, and all quotations are numbered based on order of participation |
| 30 | Data and findings consistent | Was there consistency between the data presented and the findings? | Yes |
| 31 | Clarity of major themes | Were major themes clearly presented in the findings? | Yes – See Results |
| 32 | Clarity of minor themes | Is there a description of diverse cases or discussion of minor themes? | Each theme has subthemes identified – See Results |
