# Supplementary material for: Drivers of engagement in virtual communities of practice: a qualitative study of Australian pharmacists’ perceptions and experiences
Source: Int J Clin Pharm. 2025 Apr 28;47(5):1286–95. doi: 10.1007/s11096-025-01913-3 (PMC12431881; doi:10.1007/s11096-025-01913-3)
Supplement: Supplementary file 2 — Supplementary file2 (PDF 97 KB) [file 11096_2025_1913_MOESM2_ESM.pdf]

Audit trail

Date	Time	Summary of Key Changes
18/08	9:10am	First codebook created based upon transcript 1
21/08	11:50am	15 codes added to codebook from transcript 2. All new codes added into transcript 1, with codes edited where appropriate.
22/08	10:31am	9 codes added to codebook from transcript 3. All new codes added into transcript 1 & 2, with codes edited where appropriate.
23/08	9:23am	10 codes added to codebook from transcript 4. All new codes added into transcript 1, 2 & 3, with codes edited where appropriate.
24/08	9:45am	6 codes added to codebook from transcript 5. All new codes added into transcript 1, 2, 3 & 4, with codes edited where appropriate.
24/08	3:08pm	2 codes added to codebook from transcript 6. All new codes added into transcript 1, 2, 3, 4 & 5, with codes edited where appropriate.
25/08	10:05am	Transcripts 7 and 8 coded based upon codebook
28/08	9:41am	Theme of ‘access to information’ created through:<br>Subtheme ‘learning from other pharmacists’ created through:<br>• Codes ‘sharing ideas’ and ‘helping others learn’ were condensed into code ‘differing opinions and perspectives’<br>• Codes ‘career progression’ and ‘being inspired by others career progression’ were condensed into code ‘informing career progression’<br>• Codes ‘differing opinions or perspectives,’ ‘sharing experiences’ and ‘informing career progression’ grouped<br>Subtheme ‘keeping up to date with information’ created through<br>• Codes ‘access to reliable information as members are professionals, ‘showing credentials for information credibility,’ ‘information being fact checked’ were condensed into code ‘access to reliable information’<br>• Codes ‘quick answers to questions’ and ‘consolidation of information’ were condensed into ‘easily accessible information’<br>• Codes ‘clarifying legislation changes,’ ‘access to reliable information’ and ‘easily accessible information’ grouped<br><br>Theme of ‘sense of community’ created through:<br>Subtheme ‘feeling connected to other pharmacists’ created through:<br>• Codes ‘in person networking’ and ‘online networking’ were condensed into code ‘networking opportunities’<br>• Codes ‘networking opportunities’ and ‘working together for a shared purpose’ grouped<br>Subtheme of ‘experiencing support’ created through:<br>• Codes ‘expanding number of people you can ask’ and ‘connection to local pharmacies’ were condensed into code ‘addressing professional isolations’<br>• Codes ‘opportunities to share bad experiences’ and ‘outlet to voice concerns’ were condensed into code ‘shared experiences’<br>• Codes ‘addressing professional isolations’ and ‘shared experiences’ grouped

		Theme of ‘active facilitation’ created through:<br>Subtheme of ‘facilitator filtering content’ created through:<br>• Codes ‘strict facilitation,’ ‘heavy moderation,’ ‘having a facilitator to keep things good’ were condensed and separated out into ‘facilitator ensuring relevance’ and ‘facilitator ensuring respect’<br>• Codes ‘facilitator ensuring relevance’ and ‘facilitator ensuring respect’ grouped<br>Subtheme of ‘facilitator engaging group members’ created through:<br>• Code ‘facilitator actions’ was condensed and separated out into ‘facilitator organising activties’ and ‘facilitator prompting discussion’<br>• Codes ‘facilitator organising activities’ and ‘facilitator prompting discussion’ grouped<br><br>Theme of ‘platform usability’ created through:<br>• Codes of each platform specified were analysed to determine preferable elements<br>Subtheme of ‘maintaining personal and professional boundaries’ created through:<br>• Codes ‘privacy,’ ‘encrypted platforms’ and ‘anonymous posting’ were condensed into code ‘ensuring privacy is maintained’<br>• Codes ‘platform flexibility’ and ‘flexibility’ were condensed into code ‘enabling flexibility’<br>• Codes ‘ensuring privacy is maintained’ and ‘enabling flexibility’ grouped<br>Subtheme of ‘access to online activities’ created through:<br>• Codes ‘online activities’ and ‘in person activities’ were condensed and separated into codes ‘regular meetings available to join’ and ‘access to discussion boards’<br>• Codes ‘regular meetings available to join’ and ‘access to discussion boards’ grouped<br>Subtheme of ‘easily accessible content’ created through:<br>• Codes ‘access to subgroups,’ ‘clear and professional communication,’ and ‘familiarity with platform’ grouped
