# Supplementary material for: Drivers of engagement in virtual communities of practice: a qualitative study of Australian pharmacists’ perceptions and experiences
Source: Int J Clin Pharm. 2025 Apr 28;47(5):1286–95. doi: 10.1007/s11096-025-01913-3 (PMC12431881; doi:10.1007/s11096-025-01913-3)
Supplement: Supplementary file 3 — Supplementary file3 (PDF 83 KB) [file 11096_2025_1913_MOESM3_ESM.pdf]

Code book

Codes	Definition
Differing opinions or perspectives	Accessing and considering differing points of view
Sharing experiences	Learning from others who share their experiences and having the opportunity to share one's own experiences
Informing career progression	Accessing information and networks which can assist in career progression
Clarifying legislation changes	Consolidating legislative information to gain a clearer understanding of changes
Access to reliable information	Acquiring information from reliable sources
Easily accessible information	Having the ability to access information in a way which is easy to do so
Networking opportunities	Accessing and increasing social connections
Working together for a shared purpose	Engaging in activities with a common goal in mind
Addressing professional isolations	Connecting to others in areas one has limited networks with
Shared experiences	Connecting with others who have similar experiences to oneself
Facilitator ensuring relevance	Editing content to ensure alignment with group goals
Facilitator ensuring respect	Editing content to ensure appropriate communication between group members
Facilitator organising activities	Coordinate in person and online activities for group members to engage in
Facilitator prompting discussion	Initiating conversations for members to engage in
Ensuring privacy is maintained	Having features within the platform which enable users' privacy
Enabling flexibility	Platforms being accessible to users in ways which are most convenient to them
Regular meetings available to join	Having access to participate in regular meetings
Access to discussion boards	Being able to view and participate in discussions on topics raised within the platform
Familiarity with platform	Hosting the VCOP on a platform which the user is already aware of and/or using
Clear and professional communication	Members communicating in ways which are easy to understand and reflective of their position as health professionals
Access to subgroups	Being able to view and participate in smaller, more focused groups within the wider VCOP
