# Supplementary figures and images for: Drivers of engagement in virtual communities of practice: a qualitative study of Australian pharmacists’ perceptions and experiences
Source: Int J Clin Pharm. 2025 Apr 28;47(5):1286–95. doi: 10.1007/s11096-025-01913-3 (PMC12431881; doi:10.1007/s11096-025-01913-3)

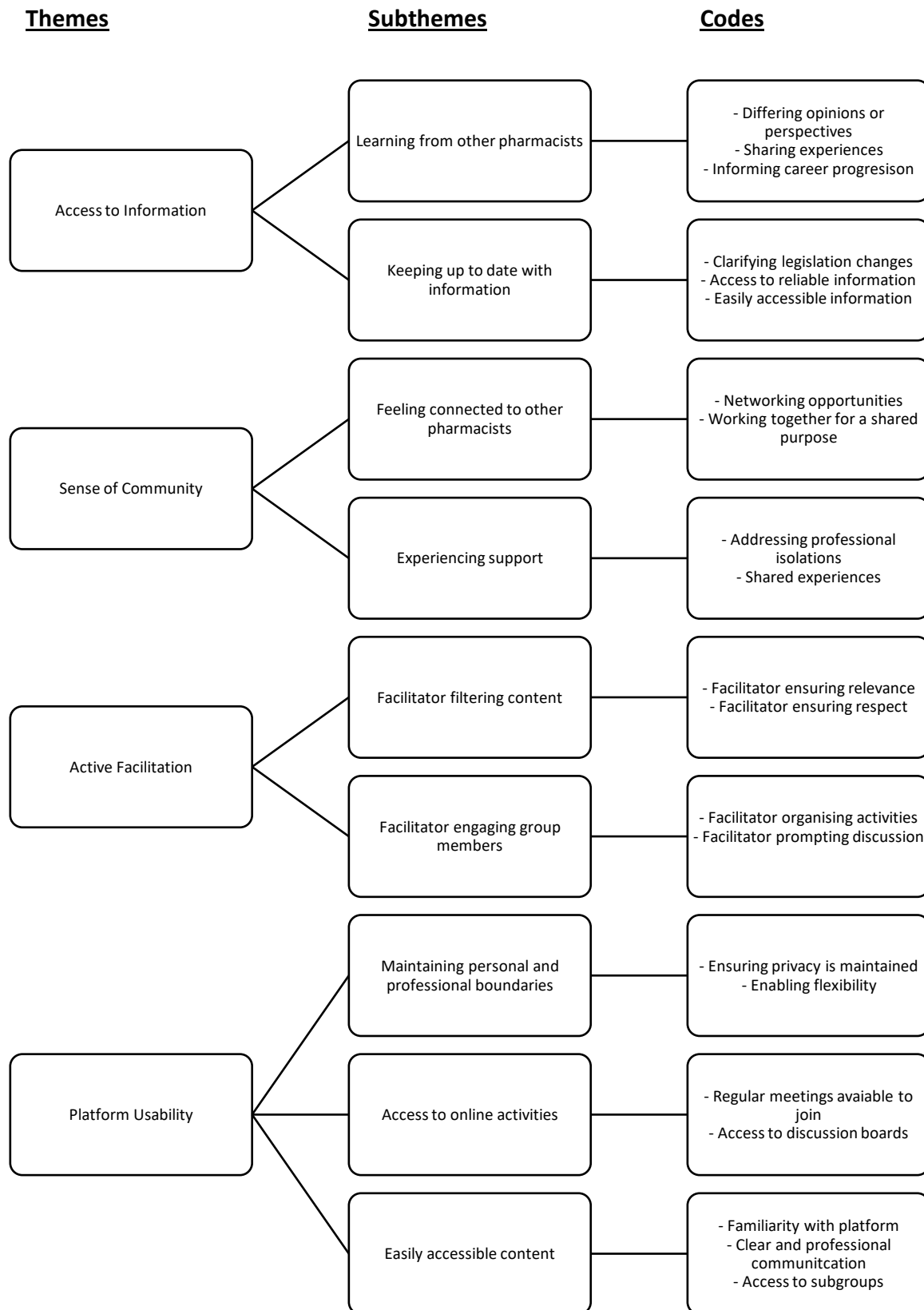

Supplement: Supplementary file 4 — Supplementary file4 (PDF 79 KB) [file 11096_2025_1913_MOESM4_ESM.pdf]
