# Supplementary material for: Drivers of engagement in virtual communities of practice: a qualitative study of Australian pharmacists’ perceptions and experiences
Source: Int J Clin Pharm. 2025 Apr 28;47(5):1286–95. doi: 10.1007/s11096-025-01913-3 (PMC12431881; doi:10.1007/s11096-025-01913-3)
Supplement: Supplementary file 5 — Supplementary file5 (PDF 99 KB) [file 11096_2025_1913_MOESM5_ESM.pdf]

Discussion Guide

Definition of virtual community of practice

Groups of people who share an interest or field of work and who come together to interact and engage in ongoing learning to improve practices. We will be talking about this in the context of online use.

Section	Primary question (necessary to ask)	Probe (not necessary to ask)	Follow up (necessary to ask)
Overview of Topic (Knowledge and experience of virtual community of practices)	Have you ever joined a pharmacy-related virtual community of practice?<br>• If yes, what did it involve? What was your experience?<br>• If no, what would you expect when joining a virtual community of practice?		
Reasons to join/motivations	If you haven't been in a virtual community of practice before, what would motivate you to join?	What would make you join a virtual community of practice?	
	If you are in a virtual community of practice, what motivated you to join?	What made you join a virtual community of practice?	
	For those who have joined before: How did you believe the virtual community of practice was going to relate to your work? (this could be day-to-day work or career progression)	How did you believe the virtual community of practice was going to correlate with your career?	
	For those who haven't joined before: How could a virtual community of practice relate to your work? (this could be day-to-day work or career progression)	How could virtual communities of practice correlate with your career?	
User satisfaction	What would a good virtual community of practice look like to you?	How would you describe a successful virtual community of practice?	What elements/components would make/have made up a good virtual community of practice?<br><br>What elements/components would you not/don't you like about them?
	What has facilitated/ do you think would facilitate good or bad communication in a virtual community of practice?	What has helped/ do you think would help good or bad communication in a virtual community of practice?	What role would leadership/facilitation/moderation play in your virtual community of practice satisfaction?

	What sort of activities have/would you be more inclined to participate in and why? I.e. Face-to-face meet ups, online meetings, posting online	Have/are there been certain activities you have been more likely to join in on and why?	
	What encourages you/would encourage you to actively participate in a virtual community of practice (such as posting, commenting, reacting, joining meetings)?	What has made/would make you actively engage in a virtual community of practice?	
	How would you find value in the virtual community of practice if you don't actively participate?	What would you gain/ have you gained from the virtual community of practice if you only viewed the content without engaging (e.g. commenting/posting)?	
Miscellaneous	What online platform would you prefer to use for a virtual community of practice? (e.g. WhatsApp/Facebook/Twitter/Discord/ Slack)	What online program would you use for a virtual community of practice?	
	Have there been any moments in your career where you have felt a stronger need to use a virtual community of practice? Any examples?	Have there been any times in your career that have you been more inclined to participate in the virtual community of practice more actively? Can you provide any examples?<br><br>For those who have joined a virtual community of practice, when in your career have you felt the need to use a virtual community of practice?	
	Would you use a virtual community of practice long-term? Why/why not?	Do you see the virtual community of practice as sustainable? Why/why not?	
